# Quality of Life and Kidney Function in Older Adults: Prospective Data of the SCOPE Study

**DOI:** 10.3390/jcm12123959

**Published:** 2023-06-09

**Authors:** Rada Artzi-Medvedik, Robert Kob, Mirko Di Rosa, Fabrizia Lattanzio, Andrea Corsonello, Ilan Yehoshua, Regina E. Roller-Wirnsberger, Gerhard H. Wirnsberger, Francesco U. S. Mattace-Raso, Lisanne Tap, Pedro G. Gil, Francesc Formiga, Rafael Moreno-González, Tomasz Kostka, Agnieszka Guligowska, Johan Ärnlöv, Axel C. Carlsson, Ellen Freiberger, Itshak Melzer

**Affiliations:** 1 Department of Nursing, The Recanati School for Community Health Professions at the Faculty of Health Sciences, Ben-Gurion University of the Negev, Beer-Sheva 8443944, Israel; rada.artzi@gmail.com; 2 Maccabi Health Services, Southern District, Omer 8496500, Israel; 3 Department of Internal Medicine-Geriatrics, Institute for Biomedicine of Aging (IBA), Friedrich-Alexander-Universität Erlangen-Nürnberg, 90408 Nürnberg, Germany; 4 Italian National Research Center on Aging (IRCCS INRCA), 60124 Ancona, Italy; 5 Department of Internal Medicine, Medical University of Graz, 8036 Graz, Austria; 6 Department of Internal Medicine, Section of Geriatric Medicine, Erasmus MC, University Medical Center Rotterdam, P.O. Box 2040, 3000 CA Rotterdam, The Netherlands; 7 Department of Geriatric Medicine, Hospital Clinico San Carlos, 28040 Madrid, Spain; 8 Geriatric Unit, Internal Medicine Department, Bellvitge University Hospital—IDIBELL—L’Hospitalet de Llobregat, 08907 Barcelona, Spain; 9 Department of Geriatrics, Healthy Ageing Research Centre, Medical University of Lodz, 90-647 Lodz, Poland; 10 School of Health and Social Studies, Dalarna University, 79188 Falun, Sweden; 11 Division of Family Medicine and Primary Care, Department of Neurobiology, Care Sciences and Society (NVS), Karolinska Institutet, 17177 Huddinge, Sweden; 12 Academic Primary Health Care Centre, Stockholm Region, 10405 Stockholm, Sweden; 13 Department of Physical Therapy, The Recanati School for Community Health Professions at the Faculty of Health Sciences, Ben-Gurion University of the Negev, Beer-Sheva 8443944, Israel

**Keywords:** quality of life, chronic kidney disease, older adults, prospective studies, cohort studies, disease progression

## Abstract

A longitudinal alteration in health-related quality of life (HRQoL) over a two-year period and its association with early-stage chronic kidney disease (CKD) progression was investigated among 1748 older adults (>75 years). HRQoL was measured by the Euro-Quality of Life Visual Analog Scale (EQ-VAS) at baseline and at one and two years after recruitment. A full comprehensive geriatric assessment was performed, including sociodemographic and clinical characteristics, the Geriatric Depression Scale-Short Form (GDS-SF), Short Physical Performance Battery (SPPB), and estimated glomerular filtration rate (eGFR). The association between EQ-VAS decline and covariates was investigated by multivariable analyses. A total of 41% of the participants showed EQ-VAS decline, and 16.3% showed kidney function decline over the two-year follow-up period. Participants with EQ-VAS decline showed an increase in GDS-SF scores and a greater decline in SPPB scores. The logistic regression analyses showed no contribution of a decrease in kidney function on EQ-VAS decline in the early stages of CKD. However, older adults with a greater GDS-SF score were more likely to present EQ-VAS decline over time, whereas an increase in the SPPB scores was associated with less EQ-VAS decline. This finding should be considered in clinical practice and when HRQoL is used to evaluate health interventions among older adults.

## 1. Introduction

The increase in the prevalence of chronic kidney disease (CKD) among older adults is related to a broad range of health concerns, including impaired physical, cognitive, and mental function [1,2,3], malnutrition [4], sarcopenia [5], and frailty [6,7]. These health-related problems among older adults increase the use of healthcare resources and challenge healthcare systems [8,9] more than other diseases [10,11,12]. This contributes to reducing the health-related quality of life (HRQoL) of community-dwelling older adults even in the early stages of CKD [13]. HRQoL was suggested to be a significant outcome, and its repeated evaluations are recommended to assess the quality of care in patients with end-stage renal disease (ESRD) [14].

HRQoL is a subjective perception of individuals or groups of their physical and mental health [15]. Not surprisingly, patients with severe CKD and ESRD show lower HRQoL scores [16,17,18,19,20]. Reduced HRQoL in patients with ESRD is associated with lower survival and higher hospitalization rates [16,17,18]. A previous cross-sectional study showed that even in early CKD stages in community-dwelling older adults, HRQoL was significantly lower compared to healthy older adults, and that the impact of CKD on HRQoL is multifactorial and partly mediated by physical performance and depressive symptoms [13]. This finding is consistent with other cross-sectional studies that have shown lower HRQoL even in the early stages (e.g., 3a and 3b) of CKD [21,22,23,24]. Longitudinal data on the association between CKD progression and HRQoL decline among older adults are rare, especially in the early stages of CKD, because this topic is less investigated. Yet, a few studies have shown that morbidity and mortality outcomes are associated with low HRQoL in patients with CKD [25,26,27,28]. In these studies, physical performance, psychological state, and HRQoL were significantly associated with increased risks of ESRD and mortality among CKD patients.

The SCOPE study represents a valuable opportunity to investigate the associations between decreased HRQoL and decline in kidney function over a two-year follow-up period in non-end-stage renal disease participants [29]. Because HRQoL is not directly affected by core symptoms of CKD, but by the deterioration in physical and psychological function [13], the aims of the present study were to investigate the association between alterations in HRQoL over a two-year period and the progression of early-stage CKD among older adults. Thus, four research questions were formulated to be addressed in the present analyses: (1) Are changes in physical functioning and depressive symptoms over a two-year period associated with decreased HRQoL? (2) Is baseline HRQoL associated with CKD progression over a two-year period? (3) Are there changes in HRQoL over a two-year period in community-dwelling older adults? (4) Is CKD progression over a two-year period associated with decreased HRQoL? The findings of this analysis will help to deepen the understanding of the impact of negative aspects of CKD on HRQoL among older adults in the early stages of the disease and to better evaluate HRQoL, not only as a consequence of the disease but also as a broad reflection of well-being among the older population.

## 2. Materials and Methods

### 2.1. Study Design and Participants

**Figure 1:**
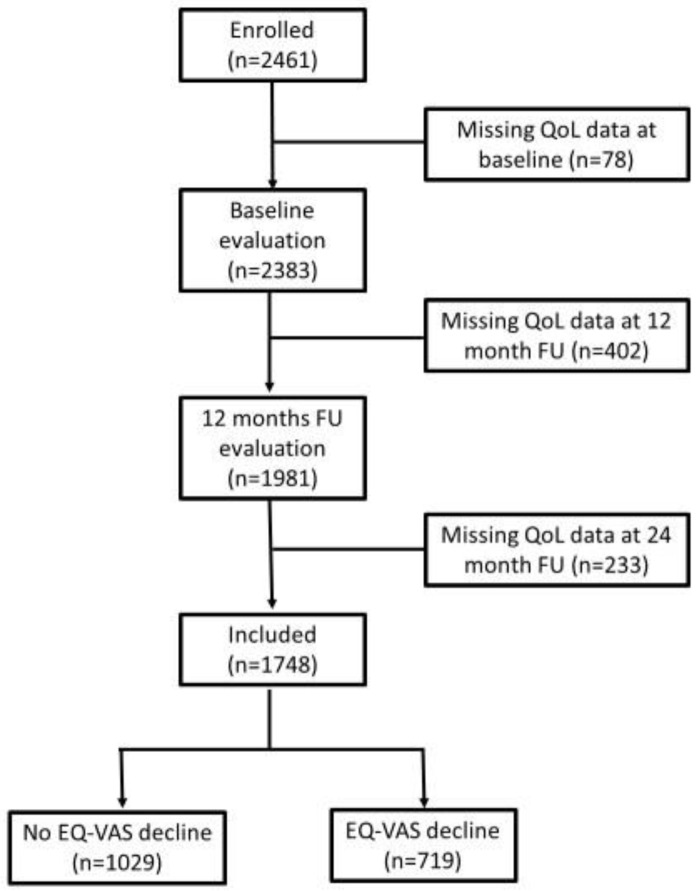
Flowchart on in- and exclusion of individuals within the current analysis.

The present analysis was performed within the framework of the “Screening for Chronic Kidney Disease among Older People across Europe” (SCOPE) project, a multicenter 2-year prospective cohort study involving people older than 75 years attending outpatient services in participating institutions in Austria, Germany, Israel, Italy, the Netherlands, Poland, and Spain (clinical trial number NCT02691546, registered on 25th February 2016 at clinicaltrials.gov). Methods of the SCOPE study have been described in detail elsewhere [29]. A full comprehensive geriatric assessment (CGA) was performed at the baseline (T0), at one year (12-month follow-up visit, T1), and 2 years (24-month follow-up visit, T2) after the recruitment. Overall, 2461 participants were initially enrolled in the study, but only 1748 provided complete HRQoL data at all three time points and were included in this analysis (Figure 1). A comparison between 1748 older adults whom we included vs. 713 who were excluded from the analysis due to incomplete HRQoL data can be found in Supplementary Table S1.

### 2.2. Study Protocol and Instruments

During face-to-face interviews, demographic and clinical variables were collected as follows: sex, age, education, marital status; blood pressure, body mass index (BMI, kg/m^2^); Mini-Mental State Examination (MMSE) [30]; history of medical conditions (e.g., diabetes mellitus, hypertension, stroke, hip fracture, chronic obstructive pulmonary disease (COPD), osteoporosis, Parkinson’s disease, anemia); the presence of lower urinary tract symptoms (LUTS) [31]; and history of falls during the last year. Overall comorbidity was assessed by the Cumulative Illness Rating Scale for Geriatrics (CIRS-G) [32] and by the number of prescribed medications taken by the participants during the past month (four or less or more than five). A 15-item Geriatric Depression Scale-Short Form (GDS-SF) [33] was used for the evaluation of self-reported symptoms of depression. Short Physical Performance Battery (SPPB) [34] and a hand grip strength test [35] were used for physical performance evaluation. Further, blood and urine laboratory tests were performed at T0, T1, and T2 and included hemoglobin, albumin, serum creatinine, urinary protein-to-creatinine ratio, and estimated glomerular filtration rate (eGFR).

### 2.3. HRQoL Assessment

HRQoL was assessed by the Euro-Quality of Life Visual Analogue Scale (EQ-VAS) and the Euro-Quality of Life 5 Dimensions (EQ-5D) [36]. The EQ-VAS asks participants to indicate their overall health on a visual analog scale, ranging from “worst possible” (score = 0) to “best possible” health (score = 100). Higher scores on this scale represent better subjective HRQoL. The EQ-5D is used to evaluate the HRQoL measure with one question on five different dimensions, which include mobility, self-care, usual activities, pain/discomfort, and anxiety/depression. The answers given to EQ-5D are scored from 1: “I have no problems…” for perfect health status, to 5: “I am unable to …” for bad health status. The 5-digit numbers for the five dimensions are combined and describe the patient’s total self-rated health status. This means that the higher the EQ-5D score on this scale, the worse HRQoL is. The EQ-VAS scores were the explanatory variable in this study. Similar to our previous cross-sectional study [13], we defined three categories of HRQoL: low as an EQ-VAS score of 0–50; intermediate as an EQ-VAS score of 51–75; and high as EQ-VAS score of 76–100.

### 2.4. HRQoL Decline Outcome

The HRQoL decline over the two-year follow-up period was defined as a downgrading of at least one category according to the EQ-VAS score (i.e., from high to intermediate or low; or from intermediate to low). Based on the above, the participants were divided into two groups: (1) the EQ-VAS decline group; and (2) the no EQ-VAS decline group. We also calculated the variation in EQ-VAS from T0 to T2 as the difference in the EQ-VAS score = Δ EQ-VAS.

### 2.5. Kidney Function Evaluation

In this analysis, eGFR was calculated using the Berlin Initiative Study (BIS) Equations (1) and (2) [37]:

Women: eGFR = (3736 × (Scr) − 0.87 × (age) − 0.95) × 0.82(1)

Men: eGFR = 3736 × (Scr) − 0.87 × (age) − 0.95,(2)

Serum creatinine was measured by Isotope Dilution Mass Spectrometry (IDMS) traceable methods. According to K/DOQI clinical practice guidelines [14], CKD is divided into 5 stages, from stage 1 with normal renal filtration rate (eGFR ≥ 90 mL/min/1.73 m^2^) to stage 5, including kidney failure or end-stage renal disease (eGFR < 15 mL/min/1.73 m^2^). For the purpose of our analysis, CKD stages 1 and 2 were combined, defined as non-CKD patients (eGFR ≥ 60 mL/min/1.73 m^2^), stages 3a + 3b defined as moderate CKD (GFR, 30–59.9 mL/min/1.73 m^2^), stage 4 defined as severe CKD (GFR, 15–29.9 mL/min/1.73 m^2^), and stage 5 defined as ESRD (GFR < 14.9 mL/min/1.73 m^2^). CKD was defined as eGFR < 59.9 mL/min/1.73 m^2^.

### 2.6. CKD Progression Outcome

CKD progression was defined as a worsening of at least one CKD stage at least once during the two-year follow-up period (i.e., a decline from CKD stages 3a + b to stages 4 or lower). Based on the above, we divided the participants into two groups: (1) CKD progression group; and (2) no CKD progression group. In addition, Δ eGFR was calculated as the difference in the eGFR from T0 to T2.

### 2.7. Physical and Mental Functions Decline Outcomes

Worsening of self-reported symptoms of depression was indicated by the positive difference in the GDS-SF score from T0 to T2 (Δ GDS-SF). Deterioration in physical functioning was assessed as the difference from T0 to T2 in the following indicators: overall SPPB score; balance score; gait speed test; chair stand test; and hand grip strength test. Negative Δ SPPB scores and Δ handgrip strength are related to declining/worsening physical function.

### 2.8. Statistical Analysis

Continuous variables were reported as a mean and standard deviation; comparisons between groups (EQ-VAS decline vs. no EQ-VAS decline) were performed by Student’s t-test or Mann–Whitney U test on the basis of their distribution (assessed using Shapiro–Wilk test). Categorical variables were expressed as absolute frequencies and percentages, and statistical differences were analyzed by Chi-square test. We first computed descriptive statistics for the subject’s characteristics at baseline and the changes over a 2-year period, i.e., from baseline (T0) to T2 (Δ). Associations between ΔEQ-VAS and variation variables (i.e., ΔeGFR, ΔSPPB—overall and subscales, and ΔGDS-SF) were assessed with Spearman rank correlation with Bonferroni-adjusted significance level and graphically represented with scatterplot diagrams. Possible interactions of co-morbidities with ΔeGFR as a factor of quality-of=life decline were also tested. Finally, three multivariable logistic models (unadjusted, age- and sex-adjusted, and fully adjusted) for each variation variable were performed to estimate their relation to EQ-VAS decline. Odds ratios (OR) and 95% confidence intervals (CI) were reported for each potential determinant. Fully adjusted models included age, gender, educational level, EQ-VAS at baseline, MMSE at baseline, BMI at baseline, GDS-SF at baseline, grip strength at baseline, and more than five prescribed medications at baseline. The variance inflation factors (VIFs) and tolerance were additionally measured in the multivariable logistic regression analysis to investigate the degree of multicollinearity among covariates: VIF >10 and tolerance <0.25 were used to define the presence of high multicollinearity (Miles J. Tolerance and variance inflation factor. First published in 29 September 2014, Wiley StatsRef Stat Ref Online, (Hoboken, New Jersey, USA); 2014. https://doi.org/10.1002/9781118445112.stat06593).

Data were analyzed using STATA version 15.1 Statistical Software Package for Windows (Stata Corp, College Station, TX, USA). Statistical significance was set a priori at *p* < 0.05.

## 3. Results

### 3.1. Participants’ Characteristics

In our cohort of 1748 older adults, 1029 (58.7%) were found with no EQ-VAS decline, and 719 (41.3%) had EQ-VAS decline (Table 1). Compared to the no EQ-VAS decline group, the older adults in the EQ-VAS decline group were more frequently females, had a higher educational level, higher BMI, lower MMSE; were taking more than five prescribed medications per day; had higher GDS-SF, higher EQ-5D, higher EQ-VAS, and lower grip strength. Interestingly, Δ GDS-SF increased in the EQ-VAS decline group vs. the no EQ-VAS decline group (+0.1 vs. −0.2, *p* = 0.046). Compared to the no EQ-VAS decline group, the EQ-VAS decline group showed a significantly higher decline in Δ SPPB balance and Δ SPPB gait speed (−0.1 vs. −0.2, *p* = 0.029 and −0.1 vs. −0.3, *p* = 0.034, respectively). No influence of Δ eGFR was found on EQ-VAS decline.

### 3.2. Associations between Δ EQ-VAS, Δ eGFR, and Other Health-Related Variables

**Figure 2:**
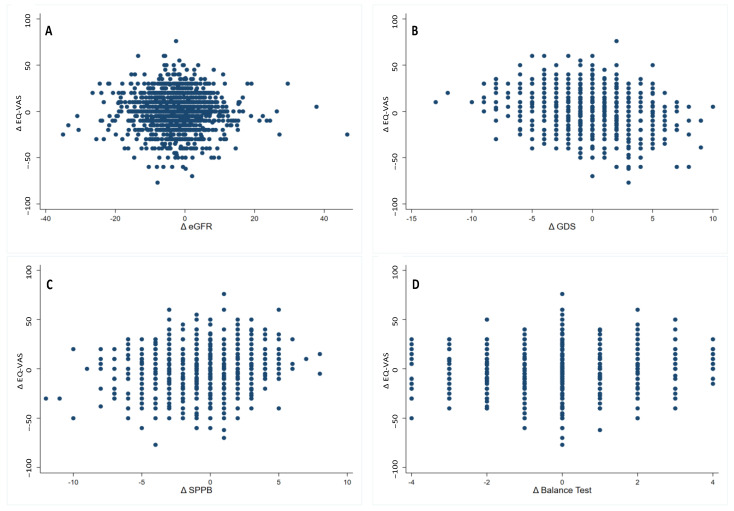
Scatterplot and linear fitted values of the difference in the EQ-VAS score from T0 to T2 (Δ EQ-VAS) and (**A**) Δ eGFR; (**B**) Δ GDS; (**C**) Δ SPPB scores; and (**D**) Δ SPPB balance scores, i.e., the difference from T0 to T2.

The association between Δ EQ-VAS score during the two-year follow-up was assessed using Spearman rank correlation with Δ eGFR, Δ SPPB score, Δ hand grip strength, and Δ GDS-SF score, as shown in Figure 2A–D. Figure 2A clearly shows no associations between Δ EQ-VAS and Δ eGFR (Rs = 0.026, *p* = 0.366). However, an increase in GDS-SF score (i.e., Δ GDS-SF) was negatively associated with Δ EQ-VAS (Rs = 0.109, *p* < 0.001, Figure 2B), suggesting that increased depressive symptoms during the two-year follow-up had a significant association with HRQoL decline among older adults. Further, changes in physical performance over time, i.e., Δ SPPB score and Δ SPPB balance score, have a low yet significantly positive association with Δ EQ-VAS (Rs = 0.096, *p* = 0.001 and Rs = 0. 0.097, *p* = 0.001, respectively, Figure 2C,D), suggesting that reduced balance during the 2-year follow-up had a significant effect on HRQoL decline among older adults. In addition, we found no association between Δ EQ-VAS and hand grip strength. Spearman’s rank correlation coefficients remained statistically significant once Bonferroni correction was applied. There was no association between co-morbidities (e.g., diabetes mellitus, hypertension, stroke, hip fracture, COPD, osteoporosis, Parkinson’s disease, anemia, LUTS, and falls history at baseline) and decline in eGFR.

According to the logistic regression analyses (Table 2), there is no contribution of a decrease in kidney function in the early stages of CKD (i.e., Δ eGFR) on EQ-VAS decline. We found that older adults who had more depressive symptoms over the two-year period (i.e., positive Δ GDS) were more likely to report having EQ-VAS decline also after adjusting for age and sex; the EQ-VAS decline was similar (OR = 1.06, 95%CI = 1.02–1.11). When adjusting for educational level, EQ-VAS at baseline, MMSE at baseline, BMI at baseline, GDS-SF at baseline, grip strength at baseline, and more than five prescribed medications at baseline contributed to a somewhat higher EQ-VAS decline (OR = 1.14, 95%CI = 1.09–1.20). Interestingly, an increase in the overall SPPB score during the two-year follow-up period was associated with less EQ-VAS decline by 5% (OR = 0.95, 95%CI = 0.91–0.99). These results were similar after adjusting for age, sex, educational level, EQ-VAS at baseline, MMSE at baseline, BMI at baseline, GDS-SF at baseline, grip strength at baseline, and more than five prescribed medications at baseline. The potential “protection effect” was even higher for Δ SPPB balance and Δ SPPB gait (OR = 0.92, 95%CI = 0.85–0.99 and OR = 0.89, 95%CI = 0.81–0.99, respectively). Adjusting for age and sex (model 2) and for educational level, EQ-VAS at baseline and MMSE at baseline (model 3) did not change the ORs. In addition, Δ SPPB chair stand and Δ hand grip revealed no association with EQ-VAS decline. As for the validity of the analyses, for Models 2 and 3, the mean VIF was <10, ranging from 1.31 and 1.41, and tolerance was >0.25 for each independent variable, confirming that no collinearity issue existed.

## 4. Discussion

In the present study, we found that 16.3% of older adults showed a decline in kidney function over the two-year follow-up period. A similar percentage of decline was found in a study conducted in the UK (18% of the participants showed a decline in kidney function within five years), whereas the risk of ESRD was very low (0.2%) [38]. However, we found a considerable decrement in HRQoL over the two-year period, whereas approximately 41% (n = 719) reported an EQ-VAS decline. Our study provides evidence that HRQoL decline among older adults is not associated with early stages of kidney function decline over a two-year period. Previous studies found that only patients with CKD on dialysis or with ESRD have a significantly lower HRQoL [39,40]. In patients with pre-dialysis chronic renal failure, the decline in HRQoL has been shown to be faster than that in the general population and was associated with an increase in serum creatinine and a decrease in hematocrit levels [25]. It has also been reported that the physical and psychological domains and HRQoL scores were significantly associated with increased risk of ESRD and mortality among CKD patients [28]. Low HRQoL across numerous subscales was independently associated with a higher risk of cardiovascular events and mortality in CKD patients, but not with CKD progression [27]. In an earlier study, an increased risk of CKD progression and mortality was associated with a lower physical health component of the SF-36 score [26]. The physical function in the above studies [26,27,28] was evaluated using an indirect measure of physical function, i.e., the physical component score of SF-12 and SF-36; thus, it was hard to compare to our cohort, in which physical function was measured using SPPB. In an earlier study [2], reduced renal function was associated with poorer physical performance (SPPB total score < 5) among older hospitalized patients with CKD, which suggests that these older hospitalized adults were more frail than in our cohort of community-dwelling independent older adults. Additionally, Tsai et al. [28] reported that 41.3% of their cohort suffered from depression compared with 14% in our cohort [13], suggesting that their cohort was less resilient. These previous findings combined with our results suggest that HRQoL decline among patients with CKD is associated with ESRD or dialysis, but not at the early stages of CKD progression.

Although earlier studies provide important findings, they are limited in scope, since they did not include older adults in the early CKD stages, which most patients with CKD belong to. More broadly, the association between HRQoL and longitudinal outcomes of physical and psychological domains among older adults in early CKD stages has not been examined. A significant body of research has investigated the possible association between HRQoL and the physical function of patients with ESRD or following kidney transplant, but these factors are not well-explored in those with less severe CKD [41]. This highlights the significance of our finding. Among the possible interpretations of our results, it is worth noting that changes in physical function and depressive symptoms over time have the strongest impact on the decline in EQ-VAS among community-dwelling elderly adults. The multivariate regression analyses showed that the decline in physical functioning and the increase in depressive symptoms over time were independently associated with HRQoL decline. Changes in physical performance over time, i.e., Δ SPPB, have a low yet significantly positive association with Δ EQ-VAS over time; these results may suggest that balance and gait function may ‘protect’ older adults from a decline in HRQoL. Another explanation might be that a better HRQoL leads to better physical function among older adults. Since our results are based on a cross-sectional study design that permits us to determine associations between variables and not causal relationships, the answer to this question should be investigated separately in a later prospective study. Most of the time, HRQoL has been interpreted as an accompanying outcome of one’s disease. However, our findings suggest that HRQoL is an important person-centered measurement, since changes in HRQoL seem to be an indicator of changes in physical and mental health status. Thus, it can be used for general population surveys, clinical research, and health policy evaluation. HRQoL also provides insight into treatment methods, since the improvement in both physical and mental function may improve HRQoL, and this may play an important role in clinical decisions and policy making. The exact mechanism underlying the association between HRQoL and the progression of CKD is abstract and difficult to explore. The decline in EQ-VAS that was found in our study is associated with reduced functional levels in the early stages of CKD among older adults, suggesting that physical or mental maintenance is not seriously addressed, which may be a risk factor for poorer HRQoL outcomes in this population. In other words, poor physical function and mental health can be surrogate parameters indicating an increase in the multi-morbidity burden that older adults feel.

The guidelines for the management of individuals with multiple coexisting chronic diseases [42] suggest that clinicians should assess physical performance such as gait speed, balance, and self-reported health status. They should also be aware of mental health issues. This is compatible with the concept of healthy aging by the World Health Organization [43], which defines healthy aging as a “process of developing and maintaining the functional ability that enables well-being in older age”. Our findings provide evidence that this approach is important in older adults even in the early stages of CKD, for which no clinical treatment is provided. Physical performance and mental state as potentially modifiable factors with an independent association with HRQoL are also important clinical considerations. When managing care for older adults, clinicians should provide care that will particularly treat physical function and mental health, since these issues affect the HRQoL of their patients. This could include planning a joint treatment with other healthcare professionals.

The strengths of our study are its meticulous protocol with a large and heterogeneous sample of respondents from different European countries and Israel. The combined use of subjective and classification-like scales reinforced analytical opportunities, as shown. It must be noted that the heterogeneous sample of older adults may also increase the variability of our outcome measures, specifically the objective measures of HRQoL. The prospective design allowed us to test the influence of physical and mental outcomes on change in HRQoL over time and to assess whether kidney function sub-stages affect HRQoL. The use of a physical performance examination such as the SPPB, which provides an objective measure of function, is also a strength of the study. However, the limitations of the current study must be noted. The main limitation of this study is the fact that our findings are based on a sample of older people who had a relatively high functional level and were in the early CKD stages, not allowing for a generalization of these conclusions to more frail older adults and ESRD patients. Second, the 713 participants who were excluded from the analysis due to incomplete information on the HRQoL may impact the results due to a selection bias. In a separate analysis, we found that the excluded older adults were older, more frequently widowed, had lower MMSE and lower SPPB at baseline, and had more frequently co-morbidities such as stroke, hypertension, diabetes, hip fractures, and depression than the 1748 individuals who were included in this study (see Supplementary Table S1). This might have affected our findings. However, it must be noted that the eGFR, depression, physical function, and handgrip strength over a two-year follow-up period, which were the main outcome parameters in the present study, were not different between groups. Finally, this observational study cannot resolve the difficulty of all hidden bias and confounding factors, despite the adjustments. Despite all these issues, longitudinal observational studies are useful for evaluating epidemiological associations and enable us to analyze the relationship between HRQoL and renal, physical, and mental outcomes through statistical techniques.

## 5. Conclusions

The findings of our study suggest that among older adults aged 75 years and older, HRQoL decline was not related to kidney function decline in the early stages of CKD. However, physical function and depressive symptoms, separately, have a low yet significant impact on HRQoL among older adults. This is an important message to clinicians and policymakers that a change in HRQoL should be taken into account to evaluate health interventions in this age group. Whether HRQoL change should be used for an evaluation depends strongly on the aims of the intervention and the characteristics of the participants. Due to the nature of this observational study, a careful interpretation of the findings as well as further research are needed. These studies are required to test whether the implementation of physical and psychological interventions in the early stages of CKD influences clinical outcomes, specifically HRQoL, among older adults.

## Figures and Tables

**Table 1:** Sociodemographic, clinical, physical, and emotional characteristics at baseline and their change over a two-year follow-up period (i.e., Δ) of: (1) older adults with no EQ-VAS decline and (2) older adults with EQ-VAS decline.

	Total	No EQ-VAS Decline	EQ-VAS Decline	*p*-Value
	N = 1748	N = 1029	N = 719	
**Baseline Assessment**				
Sex, *female* n(%)	969(55.4%)	547(53.2%)	422(58.7%)	0.022
Age, mean ± SD	79.9 ± 3.9	79.9 ± 3.9	80.0 ± 3.9	0.631
Education (years), mean ± SD	11.5 ± 4.9	11.3 ± 5.0	11.7 ± 4.9	0.026
Marital status, *widow* n(%)	553(31.6%)	312(30.3%)	241(33.5%)	0.157
BMI, mean ± SD	27.7 ± 4.3	27.4 ± 4.2	28.0 ± 4.4	0.011
MMSE, mean ± SD	28.1 ± 2.5	28.2 ± 2.5	27.9 ± 2.6	0.001
Diabetes mellitus, n(%)	398(22.8%)	227(22.1%)	171(23.8%)	0.398
Hypertension, n(%)	1314(75.2%)	770(74.8%)	544(75.7%)	0.692
Stroke, n(%)	94(5.4%)	61(5.9%)	33(4.6%)	0.222
Hip fracture, n(%)	74(4.2%)	50(4.9%)	24(3.3%)	0.120
COPD, n(%)	203(11.6%)	110(10.7%)	93(12.9%)	0.149
Osteoporosis, n(%)	534(30.5%)	317(30.8%)	217(30.2%)	0.780
Parkinson’s disease, n(%)	26(1.5%)	16(1.6%)	10(1.4%)	0.780
Anemia, n(%)	296(16.9%)	169(16.4%)	127(17.7%)	0.299
LUTS, n(%)	493(28.2%)	273(26.5%)	220(30.6%)	0.116
Falls history, n(%)	521(29.8%)	313(30.4%)	208(28.9%)	0.503
CKD, n(%)	1111(63.6%)	664(64.5%)	447(62.2%)	0.154
eGFR (mL/min/1.73 m^2^), mean ± SD	54.6 ± 14.1	54.6 ± 14.1	54.7 ± 14.1	0.605
CIRS-G score, mean ± SD	8.3 ± 4.5	8.2 ± 4.5	8.3 ± 4.4	0.624
5+ prescribed medications, n(%)	1123(64.2%)	631(61.3%)	492(68.4%)	0.004
GDS-SF score, mean ± SD	2.5 ± 2.6	2.4 ± 2.7	2.8 ± 2.4	0.000
EQ-5D, mean ± SD	7.9 ± 3.0	7.8 ± 3.1	8.0 ± 2.8	0.003
EQ-VAS, mean ± SD	72.0 ± 17.2	70.3 ± 18.4	74.5 ± 15.0	0.000
SPPB score, mean ± SD	9.0 ± 2.8	9.0 ± 2.8	9.1 ± 2.6	0.836
Balance test, mean ± SD	3.3 ± 1.1	3.2 ± 1.1	3.3 ± 1.0	0.141
Gait speed test, mean ± SD	3.3 ± 1.0	3.3 ± 1.0	3.3 ± 0.9	0.671
Chair stand test, mean ± SD	2.7 ± 1.2	2.7 ± 1.2	2.6 ± 1.2	0.227
Grip strength test, mean ± SD	25.1 ± 10.1	25.7 ± 10.2	24.1 ± 9.7	0.002
**After a two-year follow-up period**				
CKD progression, n(%)	284(16.3%)	166(16.1%)	118(16.4%)	0.190
Δ eGFR (mL/min/1.73 m^2^), mean ± SD	−0.1 ± 18.2	7.8 ± 14.4	−11.4 ± 17.0	0.360
Δ GDS-SF score, mean ± SD	−0.1 ± 2.5	−0.2 ± 2.5	+0.1 ± 2.4	0.046
Δ SPPB score, mean ± SD	−0.6 ± 2.3	−0.5 ± 2.2	−0.8 ± 2.4	0.075
Δ Balance, mean ± SD	−0.1 ± 1.2	−0.1 ± 1.2	−0.2 ± 1.2	0.029
Δ Gait speed, mean ± SD	−0.2 ± 1.0	−0.1 ± 0.9	−0.3 ± 1.0	0.034
Δ Chair stand, mean ± SD	−0.1 ± 1.1	−0.1 ± 1.1	−0.1 ± 1.2	0.512
Δ Grip strength, mean ± SD	−1.1 ± 5.5	−1.0 ± 4.9	−1.2 ± 6.2	0.837

**Abbreviations***:* EQ-VAS, Euro-Quality of Life Visual Analog Scale; BMI, body mass index; MMSE, Mini-Mental State Examination; COPD, chronic obstructive pulmonary disease; LUTS, lower urinary tract symptoms; CKD, chronic kidney disease; eGFR, estimated glomerular filtration rate; CIRS-G, Cumulative Illness Rating Scale for Geriatrics; GDS-SF, Geriatric Depression Scale-Short Form; EQ-5D, Euro-Quality of Life 5 Dimensions; SPPB, short physical performance battery. *Note:* Negative value of Δ eGFR, Δ SPPB, Δ balance, and/or Δ gait indicates a progression of CKD and physical performance, respectively, and a positive value of Δ GDS-SF indicates a progression in self-reported depressive symptoms.

**Table 2:** Determinants of EQ-VAS decline, OR (95%CI).

Independent Variable	Δ eGFR	Δ GDS-SF	Δ SPPB Total Score	Δ SPPB Balance	Δ SPPB Gait	Δ Sit to Stand	Δ Hand Grip
**Model 1.** Only independent variable	1.01 (0.99–1.02)	1.06 (1.02–1.10)	0.95 (0.91–0.99)	0.92 (0.85–0.99)	0.89 (0.81–0.99)	1.03 (0.93–1.13)	0.99 (0.97–1.01)
**Model 2.** Model 1 adjusted for age and sex	1.01 (0.99–1.02)	1.06 (1.02–1.11)	0.94 (0.91–0.99)	0.92 (0.85–0.99)	0.89 (0.80–0.98)	1.02 (0.92–1.12)	0.99 (0.97–1.01)
**Model 3.** Model 2 adjusted for educational level, EQ-VAS at baseline, MMSE at baseline, BMI at baseline, GDS-SF at baseline, grip strength at baseline, more than five prescribed medications at baseline	1.01 (0.99–1.02)	1.14 (1.09–1.20)	0.95 (0.91–0.99)	0.92 (0.84–1.00)	0.88 (0.78–0.98)	1.03 (0.93–1.14)	0.98 (0.96–1.01)

**Abbreviations:** EQ-VAS, Euro-Quality of Life Visual Analog Scale; OR, odds ratio; BMI, body mass index; MMSE, Mini-Mental State Examination; eGFR, estimated glomerular filtration rate; GDS-SF, Geriatric Depression Scale-Short Form; SPPB, short physical performance battery.

## Data Availability

Data will be available for SCOPE researchers through the project website (www.scopeproject.eu) accessed on 25 February 2016.

## Supplementary Materials

The following supporting information can be downloaded at: https://www.mdpi.com/article/10.3390/jcm12123959/s1, Supplementary Table S1.
