# Supplementary material for: Quality of Life and Kidney Function in Older Adults: Prospective Data of the SCOPE Study
Source: J Clin Med. 2023 Jun 9;12(12):3959. doi: 10.3390/jcm12123959 (PMC10299288; doi:10.3390/jcm12123959)
Supplement: Supplementary file 1 [file jcm-12-03959-s001.zip › jcm-2273255-supplementary.pdf]

Journal of
Clinical Medicine

MDPI

Table S1. Sociodemographic, clinical, physical, and emotional characteristics at baseline, and their change over a two-year follow-up period (i.e., Δ) of: 1) Older adults included in the analyses and 2) Older adults excluded from the analyses.

	**Total N=2,461**	**Included N=1,748**	**Excluded N=713**	**p-value**
Baseline assessment				
Sex, *Female* n(%)	1,380(56.1%)	969(55.4%)	411(57.6%)	0.317
**Age**, mean±SD	80.4±4.2	79.9±3.9	81.6±4.7	<0.001
Educational (years), mean±SD	11.3±4.9	11.5±4.9	10.7±4.7	<0.001
**Marital status**, *Widow* n(%)	808(32.8%)	553(31.6%)	255(35.8%)	<0.001
BMI, mean±SD	27.8±4.5	27.7±4.3	28.1±4.9	0.082
**MMSE**, mean±SD	27.9±2.7	28.1±2.5	27.3±3.0	<0.001
**Diabetes, n(%)**	605(24.6%)	398(22.8%)	207(29%)	<0.001
**Hypertension, n(%)**	1,854(75.3%)	1,314(75.2%)	540(75.7%)	<0.001
**Stroke, n(%)**	139(5.6%)	94(5.4%)	45(6.3%)	0.147
**Hip fracture, n**(%)	119(4.8%)	74(4.2%)	45(6.3%)	0.007
COPD, n(%)	287(11.7%)	203(11.6%)	84(11.8%)	0.373
Osteoporosis, n(%)	752(30.6%)	534(30.5%)	218(30.6%)	0.154
Parkinson's disease, n(%)	46(1.9%)	26(1.5%)	20(2.8%)	0.011
**Anemia, n(%)**	477(19.4%)	296(16.9%)	181(25.4%)	<0.001
LUTS, n(%)	677(27.5%)	493(28.2%)	184(25.8%)	0.692
**Falls History, n(%)**	746(30.3%)	521(29.8%)	225(31.6%)	0.009
**CKD, n(%)**	1,580(64.2%)	1,111(63.6%)	469(65.8%)	<0.001
**CIRS-F score, mean±SD**	8.5±4.6	8.3±4.5	9.2±4.9	<0.001
**5+ prescribed medications, n(%)**	1,624(66%)	1,123(64.2%)	501(70.3%)	0.004
**GDS-SF score, mean±SD**	2.7±2.7	2.5±2.6	3.1±2.9	<0.001
**SPPB score, mean±SD**	8.6±3.0	9.0±2.8	7.6±3.3	<0.001
Balance test, mean±SD	3.2±1.2	3.3±1.1	2.9±1.3	<0.001
Gait Speed test, mean±SD	3.1±1.1	3.3±1.0	2.8±1.2	<0.001
Chair Stand test, mean±SD	2.6±1.2	2.7±1.2	2.4±1.2	<0.001
Grip Strength test, mean±SD	24.4±9.8	25.1±10.1	22.4±8.9	<0.001
After two-year follow-up period				
CKD progression, n(%)	576(23.4%)	531(30.4%)	45(6.3%)	<0.001
**Δ eGFR (mL/min/1.73 m^2), mean±SD**	-2.4±6.9	-2.4±6.9	-2.3±5.7	0.717
Δ GDS-SF score, mean±SD	-0.1±2.5	-0.1±2.5	-0.1±2.7	0.910
Δ SPPB score, mean±SD	-0.6±2.3	-0.6±2.3	-1.0±3.2	0.773
Δ Balance, mean±SD	-0.2±1.2	-0.1±1.2	-0.5±1.4	0.062
Δ Gait Speed, mean±SD	-0.2±1.0	-0.2±1.0	-0.3±1.3	0.666
Δ Chair Stand, mean±SD	-0.1±1.1	-0.1±1.1	0.0±1.2	0.536
Δ Grip Strength, mean±SD	-1.1±5.5	-1.1±5.5	-1.7±6.1	0.430

Abbreviations: EQ-VAS, Euro-Quality of life visual analogue scale; BMI, Body mass index; MMSE, Mini-mental state examination; COPD, Chronic obstructive pulmonary disease; LUTS, lower urinary tract symptoms; CKD, Chronic kidney disease; eGFR, estimated glomerular filtration rate; CIRS-G, Cumulative Illness Rating Scale for Geriatrics; GDS-SF, Geriatric depression scale - Short Form; EQ-5D, Euro-Quality of life 5 dimensions; SPPB, Short physical performance battery. *Note:* negative value of Δ eGFR, Δ SPPB, Δ Balance, Δ Gait, indicates a progression of CKD and physical performance, respectively; while a positive value of Δ GDS-SF indicates a progression in self-reported depressive symptoms.
